# Severe maternal morbidity (near miss) as a sentinel event of maternal death. An attempt to use routine data for surveillance

**DOI:** 10.1186/1742-4755-5-6

**Published:** 2008-10-28

**Authors:** Maria H Sousa, Jose G Cecatti, Ellen E Hardy, Suzanne J Serruya

**Affiliations:** 1 Department of Obstetrics and Gynecology, School of Medical Sciences, University of Campinas (UNICAMP), São Paulo, Brazil; 2 CEMICAMP, Campinas, São Paulo, Brazil; 3 Department of Science and Technology, Ministry of Health, Brasília, Federal District, Brazil

## Abstract

**Background:**

To identify all the records within the Brazilian Hospital Information System (HIS) that contained information suggestive of severe maternal morbidity (near miss); to describe the diagnoses and procedures used; to identify variables associated with maternal death.

**Methods:**

A descriptive population study with data from the HIS and Mortality Information System (MIS) files of records of women during pregnancy, delivery and in the postpartum period in all the capital cities of the Brazilian states in 2002. Initially, records of women between 10 and 49 years of age were selected; next, those records with at least one criterion suggestive of near miss were selected. For the linkage of HIS with MIS and HIS with itself, a blocking strategy consisting of three independent steps was established. In the data analysis, near miss ratios were calculated with corresponding 95% confidence interval and the diagnoses and procedures were described; a multiple logistic regression model was adjusted. Primary and secondary diagnoses and the requested and performed procedures during hospitalization were the main outcome measures.

**Results:**

The overall maternal near miss ratio was 44.3/1,000 live births. Among the records indicating near miss, 154 maternal deaths were identified. The criteria of severity most frequently found were infection, preeclampsia and hemorrhage. Logistic regression analysis resulted in 12 variables, including four significant interactions.

**Conclusion:**

Although some limitations, the perspective of routinely using this information system for surveillance of near miss and implementing measures to avoid maternal death is promising.

## Background

Maternal mortality has been the target of studies in the area of public health since the beginning of the last century, particularly in the developed world, where investigations have been carried out in this field over a longer period of time. The findings of these studies have led to changes that resulted in a significant reduction in the Maternal Mortality Ratio (MMR) of these countries [1], contrary to the situation in developing countries in general [2]. It's difficult to measure the impact of changes in routine obstetrical care on maternal mortality because it is a rare event in absolute terms. Since severe maternal morbidity, also known as "near miss" is somewhat more frequent, interest has grown in using it as an indicator of the quality of obstetrical care [3]. No clear, unanimous definition has yet been developed regarding the term near miss [4]. Consequently, in addition to encouraging the study of women who survive a severe complication of pregnancy, childbirth or the postpartum period, the possibility also exists of carrying out preventive action in similar cases, thereby avoiding the fatal event by timely intervention whenever severe morbidity occurs, if it can be identified in time.

This would be especially worthwhile for developing countries. Mantel et al. [5] introduced a pilot proposal for a clinical definition of near miss, using a set of signs that are frequently present in such cases. In addition to some specific clinical data, these investigators included women admitted in intensive care units (ICU), women submitted to emergency hysterectomy and those who had suffered anesthesia accidents. Several studies have been published presenting systematic reviews on the subject [6-8]. A consensual or at least a close definition needs to be reached; however, routine information is also required that would allow severe acute maternal morbidity to be monitored and that may also serve as an alert for the introduction of timely actions to avoid an unfavorable evolution leading to death. In Brazil, the main source of healthcare information is the Ministry of Health's Information Department of the National Health System. Data from various electronic systems within Brazil are available on the Internet, including the HIS, MIS and SINASC (Information System on Liveborn Infants), that contain data of hospital records, mortality, and liveborn infant records, respectively [9]. However, these computerized data systems are known to have some limitations including incomplete coverage of cases and under-numeration of causes of maternal death. Despite that, since these systems are of public domain, such information may be used to continually monitor severe acute maternal morbidity.

The objectives of the present study were: to identify among all women admitted to hospital during pregnancy, delivery and in the postpartum period, in the 27 Brazilian capital cities in 2002, those that were indicative of severe acute maternal morbidity; to describe the diagnosis of the cause of hospitalization and the procedures carried out; to identify women who died according to the MIS, the HIS and the linkage between the two; and to identify the factors (diagnosis, procedures) associated to maternal death.

## Methods

This was a descriptive population study consisting of an analysis performed on hospital (HIS) and vital records (MIS). The sample size was calculated considering a near miss morbidity ratio of 11 events per 1,000 deliveries [5], with a sampling error of 4/1,000 and a type I error of 5%, resulting in 2,612 deliveries in each of the 27 cities studied.

The HIS-2002 and the MIS-2002 databases for each city for the year of 2002 were obtained especially for this study from the DATASUS and the Secretariat of Health Surveillance of the Ministry of Health. They contained information regarding the identification of women that enables a relationship to be established between databases. This study was approved by the Institutional Review Board of the School of Medical Sciences, University of Campinas (Letter of Approval 147/2004), and follows the principles laid out in the Declaration of Helsinki. Confidentiality was guaranteed with respect to the non-identification of cases.

The variables from the HIS used were: principal and secondary diagnoses, procedures requested and carried out, and total number of days in the ICU during hospitalization. First, the hospitalization records of women during pregnancy, delivery and in the postpartum period (diagnoses of group "O" of the ICD-10 and/or procedure beginning with 35 or 69), and with 10 to 49 years of age (reproductive age) were selected. Next, those records with at least one item suggestive of severe maternal morbidity (near miss) were selected. This second selection was based on a previously drawn-up list resulting from a search for key-words using the general criteria described by Mantel et al. [5], by Waterstone et al. [10], and by other investigators [see Additional file 1]. The ICD-10 diagnosis included in the HIS database that was supposed to be correlated with each respective criteria used to identify cases of severe maternal morbidity according to both sets were grouped. This can be understood in detail looking at the Additional file 1. This search was performed in the International Classification of Diseases (ICD-10) and also in a database containing all the codes and details of procedures accepted by the Ministry of Health in Brazil. Finally, the frequencies of all the records with diagnoses pertaining to group "O" (chapter XV of the ICD-10 corresponding to complications of pregnancy, delivery and postpartum period) and/or whose procedures belonged to the same subgroups were assessed to complete the list with the codes indicative of severity that had not been previously detected. Next, data from the 27 capital cities were separated. This proxy in defining severe maternal morbidity and/or near miss was adopted because until now there is no a clear and standard definition of what could be classified under this label by consensus.

The main fields used to establish linkage were name and date of birth. The software used to establish probabilistic record linkage between the systems was the Reclink II [11]. This freely-available program is divided into various sequential steps: standardization of databases; the linkage subdivided into blocking and matching; combination of files and clerical revision. For the linkages in the present study (HIS vs MIS and HIS vs HIS), applied separately to each one of the cities, a blocking strategy was established in multiple (three) independent steps [12]. The blocking keys used were: 1) the phonetic code (Soundex) of the first name; 2) the phonetic code of the last name; and 3) the year of birth. In the matching step, values suggested by Camargo and Coeli [12] were used to obtain the weighting factors of agreement and disagreement in order to calculate the scores.

The initial clerical revision of the junction HIS vs MIS occurred for the pairs whose agreement was not optimum (scores ≥ 10); the names were checked; if there was any doubt, the dates of birth were checked; if still necessary, the date of release from hospital according to the HIS was checked against the date of death according to the MIS. A complementary revision was also carried out for cases with lower agreement (scores between 5 and 10). A similar revision was carried out for the junction of HIS with itself (to identify cases of multiple hospitalizations of the same person).

For data analysis, the Maternal Near Miss Ratios (MNMR) were initially obtained. Next, the number of maternal deaths was listed according to the process used to identify these deaths. After this, the diagnoses and procedures were described in the selected women. Finally, multiple logistic regression analysis [13] was carried out, with stepwise-forward selection for the 19 variables and all (171) interactions between two variables, to evaluate which criteria were significantly associated with the dependent variable indicative of maternal death. The softwares used for analyses were SPSS version 11.5, and STATA version 7.0.

## Results

From a total of 634,577 medical records in the HIS files concerning women during pregnancy, delivery or in the postpartum period, and with 10–49 years of age, 5.3% of this total were initially selected, corresponding to 33,797 women whose records contained at least one item suggestive of near miss. After subtracting the maternal deaths and multiple hospitalizations identified as referring to the same woman, 32,379 women were identified as presenting severe acute maternal morbidity. The overall maternal near miss ratio (MNMR) was therefore 44.3/1,000 liveborn infants (Table 1). The lowest values of MNMR were seen in two state capitals in northern Brazil (Manaus and Boa Vista) with MNMR values of 11.8 and 12.8 near misses/1,000 liveborn infants. The highest value was seen in Teresina in northeastern Brazil (113.5 near misses/1,000 liveborn infants).

**Table 1:** Number of hospitalization records* of women during pregnancy, delivery and in the postpartum period, number of records indicative of near miss and women with near miss, number of liveborn infants and near miss morbidity ratio in the Brazilian state capital cities in 2002.

Capital cities	Number of hospitalization records (a)	Number of records suggestive of near miss (b)	Number of women with near miss (c)	Number of liveborn infants (d)	Maternal near miss ratio (MNMR) per 1000 liveborn infants [95% CI] (e)
**Region North**					
Porto Velho	5,286	388	382	7,202	**53.0 [48.0–58.5]**
Rio Branco	9,373	473	450	7,710	**58.4 [53.3–63.9]**
Manaus	36,322	455	451	38,161	**11.8 [10.8–13.0]**
Boa Vista	3,408	81	78	6,072	**12.9 [10.2–16.1]**
Belém	25,533	2,234	2,185	25,795	**84.7 [81.3–88.2]**
Macapá	9,399	378	366	8,579	42.7 [38.5–47.2]
Palmas	4,259	368	349	3,942	**88.5 [79.9–97.9]**
					
**Region Northeast**					
São Luís	21,597	1,156	1,135	18,317	**62.0 [58.5–65.6]**
Teresina	17,248	1,703	1,646	14,498	**113.5 [108.4–118.8]**
Fortaleza	44,061	2,552	2,431	39,301	**61.9 [59.5–64.3]**
Natal	12,392	520	486	13,286	**36.6 [33.5–40.0]**
João Pessoa	8,870	191	184	11,140	**16.5 [14.3–19.1]**
Recife	23,378	994	939	24,307	**38.6 [36.3–41.1]**
Maceió	17,996	999	952	16,599	**57.4 [53.9–61.0]**
Aracaju	9,185	499	490	9,354	**52.4 [48.0–57.1]**
Salvador	44,266	3,024	2,959	40,344	**73.3 [70.8–76.0]**
					
**Region Southeast**					
Belo Horizonte	28,994	1,436	1,364	32,601	41.8 [39.7–44.1]
Vitória	3,296	183	177	4,444	39.8 [34.4–46.1]
Rio de Janeiro	67,098	3,455	3,272	86,949	**37.6 [36.4–38.9]**
São Paulo	127,275	6,487	6,163	183,414	**33.6 [32.8–34.4]**
					
**Region South**					
Curitiba	20,959	1,304	1,224	26,371	46.4 [43.9–49.0]
Florianópolis	4,772	139	134	5,229	**25.6 [21.6–30.4]**
Porto Alegre	17,617	898	832	20,049	41.5 [38.8–44.4]
					
**Region Central-West**					
Campo Grande	10,983	537	513	12,347	41.6 [38.1–45.2]
Cuiabá	7,597	357	337	8,953	**37.6 [33.8–41.8]**
Goiânia	12,278	388	360	20,037	**18.0 [16.2–19.9]**
Brasília	41,135	2,598	2,520	45,799	**55.0 [52.9–57.1]**
					
**All capital cities**	634,577	33,797	32,379	730,800	44.3 [43.8–44.8]

* Source: HIS-2002;

(a) Diagnoses pertaining to the group "O" of the ICD-10 and/or procedures beginning with 35 or 69;

(b) Contained in the previous column (corresponds to 5.3% of the same);

(c) Contained in the previous column (maternal deaths and records of multiple hospitalization of the same person have been excluded);

(d) Source: SINASC-2002;

(e) Values in bold are significantly different from the mean country.

One hundred and fifty-four maternal deaths were identified among the medical records suggestive of near miss. Of these deaths, 87% (48% plus 39%) were found using the linkage of the HIS with the MIS, and 61% (48% plus 13%) from the "hospital charges" field of the HIS. Therefore, using the two procedures together, it was possible to identify 48% of these deaths (Table 2).

**Table 2:** Number of records of maternal deaths identified in the selection of cases of near miss according to geographical region, and the procedure used for localization.

	Procedure for localizing deaths		
	
Capital cities in the region:	Using *linkage *with MIS and the "hospital charges" field	Using only *linkage *with MIS	Using only the "hospital charges" field of the HIS
North	10	7	2
Northeast	16	12	5
Southeast	35	30	8
South	6	2	0
Central-West	7	9	5
			
Total	74	60	20
(%) *	(48%)	(39%)	(13%)

* Percentage in relation to the total of 154 maternal deaths identified.

The descriptive analysis of diagnoses and procedures showed that the most frequent diagnostic criteria for maternal death were immunological disorders/severe sepsis with 20.8% of the cases, followed by severe preeclampsia (14.9%) and severe hemorrhage (14.3%). For the cases of near miss, the same main criteria of signs and symptoms applied: severe preeclampsia (30.6%), immunological disorder/severe sepsis (23.7%) and severe hemorrhage (20.3%). For the procedures, the corresponding percentages were, in general, lower and the item concerning admission to the ICU, in which diagnoses did not appear, was the most frequent criterion for maternal death, with 47.4% of 154 cases; however, it was the fourth most frequent criterion for describing cases of near miss (Table 3).

**Table 3:** Percentage of records according to criteria used for diagnoses and procedures in cases of near miss and maternal death (HIS, 2002)

	Diagnoses		Procedures	
	
Group/Criterion; item	Near miss (%)	Maternal death (%)	Near miss (%)	Maternal death (%)
**A. Mantel et al., 1998**				
Cardiac disorder	5.5	8.4	1.3	1.3
Vascular disorder	< 0.1	1.9	--	--
Immunological disorder^&^	23.7	20.8	8.9	14.9
Respiratory disorder	0.3	0.6	< 0.1	0.6
Kidney disorder	0.5	1.3	< 0.1	0.6
Liver disorder	< 0.1	0.0	0.0	0.0
Metabolic disorder	0.1	0.0	< 0.1	0.0
Coagulation disorder	2.1	2.6	< 0.1	0.0
Cerebral disorder	< 0.1	1.3	< 0.1	0.6
Admission to ICU	--	--	3.4 ^#^	47.4 ^#^
Emergency hysterectomy	--	--	0.4	7.8
Anesthesia accident	0.1	0.0	--	--
				
**B. Waterstone et al., 2001**				
Severe preeclampsia	30.6	14.9	9.3	9.7
Eclampsia	7.0	11.0	1.1	5.2
HELLP syndrome	--	--	--	--
Severe hemorrhage	20.3	14.3	5.5	5.2
Severe sepsis ^&^	23.7	20.8	8.9	14.9
Uterine rupture	0.6	0.0	--	--
				
**C. Others**				
Acute abdomen	0.4	1.3	--	--
HIV-related disease	0.1	1.3	--	--
Other surgical procedures	--	--	1.8	5.2
				
Total	(32,379)	(154)	(32,379)	(154)

-- No codes related to the problem

^&^Definition identical to items "Immunological disorder" and "Severe sepsis"

^# ^Not found among the 6 procedures of the ICU diary; however found in the field referring to the total number of days in the ICU during hospitalization.

Logistic regression analysis resulted in 12 variables in the final model, among them four significant interactions: admission to the ICU with severe infection, severe preeclampsia with severe hemorrhage, emergency hysterectomy with severe hemorrhage, and admission to the ICU with severe hemorrhage. With the adjusted equation of the final model, different probabilities were calculated, the highest of them being that observed for the criterion of admission to the ICU with severe infection (0.3537), while the lowest probability (0.0015) was for the individual criterion of severe preeclampsia (Table 4).

**Table 4:** Coefficient, standard error and significance of the variables of the final model of logistic regression analysis; estimated equation for calculating the probabilities and some examples calculated [n = 32,533]

**Variables**	**Coefficient**	**Standard error of the coefficient**	**p**
Admission to ICU with severe infection	3.1320	0.4564	< 0.0001
Admission to ICU	2.5677	0.2283	< 0.0001
Severe preeclampsia with severe hemorrhage	4.6209	0.6360	< 0.0001
Cerebral disorder	3.1013	0.7710	< 0.0002
HIV-related disease	2.5265	0.7502	0.0008
Emergency hysterectomy with severe hemorrhage	3.1746	0.8221	< 0.0002
Severe preeclampsia	-1.0093	0.2669	< 0.0005
Admission to ICU with severe hemorrhage	-2.3838	0.9754	0.0145
Vascular disorder	2.8264	0.7983	< 0.0005
Severe infection	-0.8317	0.3202	0.0094
Emergency hysterectomy	0.6534	0.5433	0.2292
Severe hemorrhage	-0.6591	0.3143	0.0360
Constant	-5.4710	0.1912	< 0.0001
			
**Estimated equation**:			
Prob(death) = 1/(1+exp(-(-5.4710 +3.1320 × Int(ICU, Infection) + 2.5677 × ICU + 4.6209 × Int(Preeclampsia, Hemorrhage) + 3.1013 × Cerebral disorder + 2.5265 × HIV + 3.1746 × Int(Hysterectomy, Hemorrhage) - 1.0093 × Preeclampsia + -2.3838 × Int(ICU, Hemorrhage) + 2.8264 × Vascular disorder - 0.8317 × Infection + 0.6534 × Hysterectomy - 0.6591 × Hemorrhage)))			
			
**Examples**:	**Prob (death)**		
Admission to ICU with severe infection	0.3537		
Emergency hysterectomy with severe hemorrhage	0.0910		
Cerebral disorder	0.0855		
Severe preeclampsia with severe hemorrhage	0.0746		
Vascular disorder	0.0663		
Admission to ICU	0.0520		
HIV-related disease	0.0500		
Emergency hysterectomy	0.0080		
Admission to ICU with severe hemorrhage	0.0026		
Severe hemorrhage	0.0022		
Severe infection	0.0018		
Severe preeclampsia	0.0015		

## Discussion

The general results of the current study showed that overall maternal near miss ratio for the state's capitals in Brazil was 44.3/1,000 live births. Among the records classified as near miss, 154 maternal deaths were identified. The criteria of severity most frequently found were infection, preeclampsia and hemorrhage. They also allow having some probabilities of dying according to their combined occurrence, enhancing the perspective of using this routine information system for surveillance of near miss.

There are various advantages to using information routinely collected by the HIS of the Brazilian National Health Service, among them the low cost and relatively short period of time between collection and the availability of the files for public use [14]. Nevertheless, some limitations are known to exist. The first concerns the very conception of this system, which was designed for managerial purposes, to regulate the system, and to pay for hospitalizations; not for research or surveillance purposes. In a recent publication regarding the scientific production that has been carried out using data from the HIS in several fields of study [15], the authors emphasize that, despite incomplete coverage and problems with respect to the reliability of the information contained in this system, the results of some studies have been found to be coherent with current knowledge, thereby indicating its importance and potential for use in research.

In the present study, some problems could be identified. Initially, the intention was to use the number of deliveries as the denominator to calculate the maternal near miss ratio. However, due to the fact that this figure could not be directly obtained from the database, the number of liveborn infants coming from the Information System on Liveborn Infants (SINASC), was considered instead. SINASC includes a subset of births occurring in private hospitals that are not part of the National Health System, which is not the case of the HIS. In addition, the total number of deliveries should be greater than the number of liveborn infants since the former includes stillborn infants. Therefore, it was necessary to use the number of liveborn infants for the calculation of the MNMR, which consequently resulted in the higher values obtained.

The maternal near miss ratio in this study may be overestimated due to the limitation in the lower denominator that was used (number of liveborn infants instead of deliveries). On the other hand, it should be emphasized that the use of data deriving from the HIS is restricted to hospitalizations with details limited to two diagnoses and two procedures; therefore, the MNMR of 44/1,000 liveborn infants may be underestimated. From the set of cases selected, it was possible to note that the most frequent diagnoses and procedures found in cases of maternal death were also those identified in cases of near miss, i.e. infection, preeclampsia and hemorrhage.

Another problem was detected when approximately half the maternal deaths declared in the MIS could not be found in the HIS, another aspect of this study that has already been reported [16]. In the present paper, when analysis was based on the HIS, only 61% of the maternal deaths that were found in the selection of near miss cases would have been identified by the system itself using the "hospital charges" field. In this case, it was important to link the two systems using the Reclink program [11]; however, information regarding identification (such as full name), which is not normally available, had to be obtained, making it a difficult matter due to issues of data confidentiality.

By classifying the women into two groups (near miss or maternal death), the principal items of diagnoses and procedures were able to be described according to the tentative criteria suggested by Mantel et al., 1998 [5], by Waterstone et al., 2001 [10] and some other criteria [17], and it was possible to observe that the most frequent criteria were those referring to signs and symptoms. These results show a similar path in the identification of cases of near miss to that followed by Canada, the only country in the world, as far as we are aware, to systematically collect and analyze such information in an attempt to generate more subsidies for preventive actions related to maternal deaths [17]. In this specific situation, as well as a list of criteria based on diagnoses also coded in the ICD, Canada also uses its own list of procedures related to the severity of the maternal condition in much the same way as we have used in the present study. Using the Canadian national databases of vital and hospital records, an NMMR of 4.62/1000 deliveries was calculated [17], a figure 10 times lower than that found in this study despite the differences in the definition of cases and also in the denominator used. However, this proposal of using routine data from HIS is basically for identifying only cases of severe maternal morbidity, considering the mortality is much better recorded and more easily identified in the specific MIS from the Ministry of Health.

In the final logistic regression analysis, it was possible to calculate an equation for estimating the probability of dying. Considering the three groups of criteria, referred to as management complexity, organic dysfunction and signs and symptoms [4,8], the present study showed a combination of these items, with respect to the criteria established by Mantel et al. [5] and Waterstone et al. [10]. This is in agreement with the World Health Organization's recommendations for the study and surveillance of severe maternal morbidity [4] and the possibility of really using routine data from information systems as a way to identify these cases [18]. This would facilitate to intervene in time to change the clinical course of the complication, thus avoiding a maternal death or an even more serious sequelae, within a true, retro-feeding surveillance system using the information from the system itself. It should be emphasized that currently the Brazilian hospital information system is based on the ICD-10 list of diagnosis, what was used to identify cases of severe maternal morbidity. When a standard definition to be adopted by WHO is available, hopefully we could check its validity on identifying cases of severe maternal morbidity and also those who finished with death.

The probabilities calculated here highlight some problems that merit particular vigilance, such as hospitalization in the ICU in association with infection, emergency hysterectomy with severe hemorrhage, and cerebral disorder, among others. Despite the limitations that exist in the HIS, the prospect of using this data source routinely seems viable. If the usefulness of this type of surveillance system is confirmed, the resulting advantages to women's health in developing countries would be enormous if similar systems could be recommended and replicated as a strategy for safe motherhood.

## Conclusion

Although some limitations, the perspective of routinely using this information system for surveillance of near miss and implementing measures to avoid maternal death is promising.

## Abbreviations

HIS: Hospital Information Systeml ICD: International Classification of Diseases; ICU: intensive care unit; MIS: Mortality Information System; MMR: Maternal Mortality Ratio; MNMR: Maternal near miss ratio; SINASC: Information System on Liveborn Infants.

## Competing interests

The authors declare that they have no competing interests.

## Authors' contributions

MHS and JGC participated in all steps of the study, including research planning, data collection, analysis and writing the manuscript. EEH and SJS participated in the project planning and review of the manuscript. All authors gave suggestions, read the manuscript carefully, fully agreed on its content and approved its final version.

## Supplementary Material

Additional file 1 Click here for file
