# Supplementary material for: Severe maternal morbidity (near miss) as a sentinel event of maternal death. An attempt to use routine data for surveillance
Source: Reprod Health. 2008 Oct 28;5:6. doi: 10.1186/1742-4755-5-6 (PMC2583985; doi:10.1186/1742-4755-5-6)
Supplement: Additional file 1 [file 1742-4755-5-6-S1.doc]

**APPENDIX A**

Diagnosis (ICD-10) and procedures used for defining near miss maternal morbidity, according to the Mantel et al. (1998), Waterstone et al. (2001) and other extra criteria

| Criteria/  Markers | General characterization of diagnoses [ICD-10 codes] | General characterization of procedures |
| --- | --- | --- |
|  |  |  |
| **A. Mantel et al., 1998** |  |  |
|  |  |  |
| A.1 Organ system-based |  |  |
|  |  |  |
| 1. Cardiac dysfunction |  |  |
| 1.1 Pulmonary oedema  1.2 Cardiac arrest | - Pulmonary edema [**J81**]  - Cardiomyopathy; congestive heart disease [**I11.0**; **I42.0**; **I42.1**; **I42.8**;  **I42.9**; **I43.8**; I46; I46.0; I46.9; **I50.0**; **I50.1**; **I50.9**; **O75.4**; **O90.3**; R57.0] | - Acute pulmonary edema; Pulmonary embolism or infarction  - Cardiac insufficiency; Hypertensive crises; Congenital  cardiopathy with cardiac insufficiency; Arrhythmia;  Cardiovascular shock; Malignant hypertension |
|  |  |  |
| 2. Vascular dysfunction  2.1 Hypovolaemia requiring blood | - Hypovolemic shock; volume depletion [**E86**; **O75.1**; **R57.1**; **R57.9**;  T81.1] |  |
|  |  |  |
| 3. Immunological dysfunction  3.1 Intensive care admission for  sepsis | - Infection; Sepsis; Abortion complicated by genital tract infection;  Peritonitis; Salpingitis [A02.1; A22.7; A26.7; A32.7; A40; **A40.0**; A40.1; A40.2; A40.3; A40.8; A40.9; A41; A41.0; **A41.1**; **A41.2**; **A41.3**; A41.4; **A41.5**; **A41.8**; **A41.9**; A42.7; A54.8; B37.7; **K35.0**; **K35.9**; **K65.0**; **K65.8**; K65.9; **M86.9**; **N70.0**; **N70.9**; **N71.0**; **N73.3**; **N73.5**; **O03.0**; **O03.5**; **O04.0**; **O04.5**; **O05.0**; **O05.5**; **O06.0**; **O06.5**; **O07.0**; **O07.5**; **O08.0**; **O08.2**; **O08.3**; **O41.1**; **O75.3**; **O85**; O86; **O86.0**; **O86.8**; O88.3; T80.2] | - Post cesarean wound infection; Infection of delivery and puerperium; Sepsis; Acute adnexal infection; Post-cesarean peritonitis; Peritonitis |
| 3.2 Emergency hysterectomy for  sepsis |
|  |  |  |
| 4. Respiratory dysfunction  4.1 Intubation and ventilation for  more than 60 min  4.2 Oxygen saturation <90% lasting  more than 60 min  4.3 Ratio Pa O2/ Fi O2 ≤ 3 | - Respiratory failure; Respiratory arrest; Pulmonary embolism; Abortion complicated by embolism [**I26.9**; **J80**; J96; **J96.0**; J96.9; **O03.7**; **O04.7**; **O05.2**; **O06.2**;**O06.7**; **O88.1**; **R09.2**] | - Acute respiratory failure |
|  |  |  |
| 5. Renal dysfunction  5.1 Oliguria defined as <400 ml/24h | - Renal failure following ectopic and molar pregnancy [**O08.4**; R34] |  |
| 5.2 Acute deterioration of urea to  >15 mmol/l or of creatinine to  >400 mmol/l | - Acute renal failure [E72.2; I12.0; I13.1; I13.2; N17; N17.0; N17.1;  N17.2; **N17.8**; **N17.9**; **N18.0**; **O08.4**; **O90.4**] | - Acute renal failure |
|  |  |  |
| 6. Liver dysfunction  6.1 Jaundice in the presence of pre-  eclampsia | - Liver disorders; Viral hepatitis complicating pregnancy, childbirth and the puerperium [K72; K72.0; K72.9; **O26.6**; **O98.4**] | - No one procedure found |
|  |  |  |
| 7. Metabolic dysfunction  7.1 Diabetic keto-acidosis | - Diabetes mellitus with coma or ketoacidosis [**E10.0**; **E10.1**; E11.0; **E11.1**; E12.0; E12.1; E13.0; E13.1; E14.0; **E14.1**] |  |
| 7.2 Thyroid crisis | - Thyrotoxicosis; Metabolic disorders following an ectopic and molar  pregnancy [E05; **E05.0**; E05.1; E05.2; E05.3; E05.4; E05.5; **E05.8**; **E05.9**; E06.0; E07; E07.8; **E07.9**; **O08.5**] | - Thyroid dysfunction; Thyrotoxicosis |
|  |  |  |
| 8. Coagulation dysfunction  8.1 Acute thrombocytopenia  requiring platelet transfusion | - Disseminated intravascular coagulation; Coagulation defect [**D65**; D68; **D68.9**; D69.4; D69.5; D69.6; D82.0; **O45.0**; **O72.3**] | - Idiopathic thrombocytopenic purpura |
|  |  |  |
| 9. Cerebral dysfunction  9.1 Coma lasting > 12 h |  |  |
| 9.2 Subarachnoid or intracerebral  haemorrhage | - Intracerebral hemorrhage; Cerebrovascular accident; Cerebral venous  thrombosis in pregnancy [**G93.6**; I60; I60.0; I60.1; I60.2; I60.3; I60.4;  I60.5; I60.6; I60.7; I60.9; I61; I61.0; I61.1; I61.2; I61.3; I61.4; I61.5;  I61.6; **I61.8**; **I61.9**; **I64**; I69.1; **O22.5**] | - Conservative management of cerebral bleeding |
| A.2 Management-based |  |  |
|  |  |  |
| 10. Intensive care admission  10.1 For any reason |  | - Total number of days at ICU during hospitalization (variable “Uti_mes3”*) |
|  |  |  |
| 11. Emergency hysterectomy  11.1 For any reason |  | - Total or subtotal hysterectomy; Hysterectomy with uni or bilateral ooforectomy; Puerperal hysterectomy |
|  |  |  |
| 12. Anaesthetic accidents  12.1 Severe hypotension associated  with a spinal or epidural anaesthetic | - Pulmonary complications of anesthesia during pregnancy, labor and  delivery [O29; **O29.0**; O29.1; O29.2; O29.3; O29.5; O29.8; **O29.9**; O74; O74.0; **O74.1**; O74.2; O74.3; O74.4; O74.6; O74.8; O74.9; O89; **O89.0**; O89.1; **O89.2**; **O89.3**; **O89.5**; O89.8; **O89.9**; T88.2; T88.3; T88.5] |  |
| 12.2 Failed tracheal intubation  requiring anaesthetic reversal |
|  |  |  |
| **B. Waterstone et al., 2001** |  |  |
|  |  |  |
|  |  |  |
| 1. Severe pre-eclampsia | - Mild, severe or unspecified pre-eclampsia; Pre-existing hypertensive disorder with superimposed proteinuria [**O11**; **O14.0**; **O14.1**; **O14.9**] | - Severe pre-eclampsia |
|  |  |  |
| 2. Eclampsia | - Eclampsia in pregnancy, labor or delivery [O15; **O15.0**; **O15.1**; **15.2**; **O15.9**] | - Labor with eclampsia; Eclampsia |
|  |  |  |
| 3. HELLP syndrome |  |  |
|  |  |  |
| 4. Severe haemorrhage | - Incomplete abortion complicated by delayed or excessive hemorrhage;  Placenta previa with hemorrhage; Premature separation of placenta [**D62**; **O03.1**; **O03.6**; **O04.1**; **O04.6**; **O05.1**; O05.6; **O06.1**; **O06.6**; **O07.1**; **O07.6**; **O08.1**; **O44.1**; **O45.0**; **O45.8**; **O45.9**; O46; **O46.0**; **O46.8**; **O46.9**; **O67.0**; **O67.8**; **O67.9**; O69.4; O72; **O72.0**; **O72.1**; **O72.2**] | - Hemorrhage in pregnancy |
|  |  |  |
| 5. Severe sepsis | - Infection; Sepsis; Abortion complicated by genital tract infection;  Peritonitis; Salpingitis [A02.1; A22.7; A26.7; A32.7; A40; **A40.0**; A40.1; A40.2; A40.3; A40.8; A40.9; A41; A41.0; **A41.1**; **A41.2**; **41.3**; A41.4; **A41.5**; **A41.8**; **A41.9**; A42.7; A54.8; B37.7; **K35.0**; **K35.9**; **K65.0**; **K65.8**; K65.9; **M86.9**; **N70.0**; **N70.9**; **N71.0**; **N73.3**; **N73.5**; **O03.0**; **O03.5**; **O04.0**; **O04.5**; **O05.0**; **O05.5**; **O06.0**; **O06.5**; **O07.0**; **O07.5**; **O08.0**; **O08.2**; **O08.3**; **O41.1**; **O75.3**; **O85**; O86; **O86.0**; **O86.8**; O88.3; T80.2] | - Post cesarean wound infection; Infection of delivery and puerperium; Sepsis; Acute adnexal infection; Post-cesarean peritonitis; Peritonitis |
|  |  |  |
| 6. Uterine rupture | - Rupture of uterus before or during labor; Disruption of cesarean delivery wound [**O71.0**; **O71.1**; **O90.0**] |  |
|  |  |  |
| **C. Others** |  |  |
|  |  |  |
|  |  |  |
| 1. Acute abdomen | - Acute abdomen [**R10.0**] |  |
|  |  |  |
| 2. HIV disease | - Human immunodeficiency virus disease resulting in infections diseases [**B20**; **B20.0**; **B20.1**; **B20.4**; **B20.8**; **B20.9**] |  |
|  |  |  |
| 3. Others surgical procedures |  | - Multiple surgical procedures; Exploratory laparotomy;  Laparotomy for uterine suture; Surgical treatment for post partum acute uterine inversion |
|  |  |  |

The bolded codes for diagnosis were found in the SIH file for Brazilian capitals in 2002;

The characterization and codification of item 3 of Mantel et al. (1998) is the same used for item 5 of Waterstone et al. (2001).

* Specific variable from database that informs the total number of days at ICU during hospitalization.
